# The Global Burden of Osteoporosis, Low Bone Mass, and Its Related Fracture in 204 Countries and Territories, 1990-2019

**DOI:** 10.3389/fendo.2022.882241

**Published:** 2022-05-20

**Authors:** Yuyan Shen, Xin Huang, Junyun Wu, Xiling Lin, Xiao Zhou, Zhiang Zhu, Xiaowen Pan, Jingya Xu, Jie Qiao, Tianyue Zhang, Linxia Ye, Hongwei Jiang, Yuezhong Ren, Peng-Fei Shan

**Affiliations:** ^1^Department of Endocrinology and Metabolism, the Second Affiliated Hospital of ZheJiang University School of Medicine, Hangzhou, China; ^2^Endocrine and Metabolic Disease Center, The First Affiliated Hospital, and College of Clinical Medicine of Henan University of Science and Technology, Luoyang, China; ^3^Medical Key Laboratory of Hereditary Rare Diseases of Henan, Luoyang, China; ^4^Luoyang Sub-Center of National Clinical Research Center for Metabolic Diseases, Luoyang, China; ^5^Binjing Institute of Zhejiang University, Hangzhou, China

**Keywords:** low bone mineral density, osteoporosis, fracture, death, disability-adjusted life years (DALYs), global burden

## Abstract

**Background:**

Low bone mineral density (LBMD), including osteoporosis and low bone mass, has becoming a serious public health concern. We aimed to estimate the disease burden of LBMD and its related fractures in 204 countries and territories over the past 30 years.

**Methods:**

We collected detailed information and performed a secondary analysis for LBMD and its related fractures from the Global Burden of Disease Study 2019. Numbers and age-standardized rates related to LBMD of disability-adjusted life-years (DALYs) and deaths in 204 countries and territories were compared by age, gender, socio-demographic index (SDI), and location.

**Results:**

Global deaths and DALYs number attributable to LBMD increased from 207 367 and 8 588 936 in 1990 to 437 884 and 16 647 466 in 2019, with a raise of 111.16% and 93.82%, respectively. DALYs and deaths number of LBMD-related fractures increased 121.07% and 148.65% from 4 436 789 and 121248 in 1990 to 9 808 464 and 301 482 in 2019. In 2019, the five countries with the highest disease burden of DALYs number in LBMD-related fractures were India (2 510 288), China (1 839 375), United States of America (819 445), Japan (323 094), and Germany (297 944), accounting for 25.59%, 18.75%, 8.35%, 3.29%, and 3.04%. There was a quadratic correlation between socio-demographic index (SDI) and burden of LBMD-related fractures: DALYs rate was 179.985-420.435SDI+417.936SDI^2^(R^2 =^ 0.188, p<0.001); Deaths rate was 7.879-13.416SDI+8.839 SDI^2^(R^2 =^ 0.101, p<0.001).

**Conclusions:**

The global burden of DALYs and deaths associated with LBMD and its related fractures has increased significantly since 1990. There were differences in disease burden between regions and countries. These estimations could be useful in priority setting, policy-making, and resource allocation in osteoporosis prevention and treatment.

## Introduction

Low bone mineral density (LBMD), including osteoporosis and low bone mass, is a chronic bone metabolic disease characterized by impaired bone mass and microstructure, leading to increased risk of fractures in various parts of the body. This public health problem has brought a heavy burden to the global economic, social and health development (1, 2). Osteoporosis currently affects more than 10 million people in the United States and is expected to affect approximately 14 million adults over the age of 50 by 2020. Worldwide, about 200 million women suffer from osteoporosis (1, 3).

It is important to note that the most serious complication of osteoporosis is fracture. It was projected that by 2050, the worldwide incidence of hip fracture in men would increase by 310% and 240% in women (4). Results from large prospective studies show that almost all types of fractures increase in patients with LBMD, and that adults who already have one type of fracture are 50% to 100% more likely to have a different type of fracture, regardless of the type (5, 6). The concealment and particularity of osteoporosis are that the osteoporotic population usually lacks clinical symptoms prior to the fracture event, thus fragility fracture becomes the dominant clinical presentation. About one in three women and one in five men, typically aged 50 and older, experience a fragility fracture in the rest of their lives (7, 8). In Europe, fragility fractures are the fourth leading cause of chronic diseases, behind ischemic heart disease, dementia and lung cancer (9). Moreover, older people with osteoporosis are at increased risk for persistent fragility fractures, and factors such as falls can accelerate it (10). The aging of the world’s population and changing lifestyles will lead to rising rates of chronic diseases, such as osteoporosis (11, 12). There was a continuous relationship between decreased bone mineral density and increased fracture risk, with a significant increase in fracture risk for each 1SD decrease in bone mineral density (13). Osteoporotic fractures will not only cause pain to individuals, such as deformity and pain, resulting in physical damage and serious psychological disorders such as depression, anxiety and fear, but also cause huge economic pressure to the society (14–17).

To our knowledge, there is few of global data on the disease burden associated with LBMD and fragility fractures. In the Global Burden of Disease Study 2019 (GBD 2019), LBMD is a risk factor to assess its impact on human health and longevity. In GBD 2019, death and health loss from osteoporotic fractures cannot be directly identified because as cause of death data from vital registration and verbal autopsy attribute injury deaths to causes of death (e.g., falls or road injury) and not nature of injury (such as fractures) (18, 19). However, GBD 2019 restricted assessment of the health burden of LBMD to a list of causes that were deemed to cause fractures: falls, pedestrian road injuries, motor vehicle road injuries, motorcyclist road injuries, other exposure to mechanical forces, other transport injuries, cyclist road injuries, physical violence by other means, non-venomous animal contact and other road injuries (20, 21). Most above events can directly result in fractures because of injuries and violence, not LBMD. As this has been proven in previous articles, most osteoporotic fractures limited to falls are expected to be coded (20). Therefore, in this study only disease burden of LBMD-related falls was considered as osteoporotic fractures. We used GBD 2019 to capture data of LBMD and LBMD-related falls on deaths and DALYs as absolute numbers and age-standardized rates for all age groups and 204 countries and territories annually from 1990 to 2019 to investigate the trend of the burden of LBMD and osteoporotic fractures, and to provide evidence for the adjustment of health resources and policies (22).

## Methods

### Overview

The Global Health Data Exchange is the world’s most comprehensive survey to date, covering census, household surveys, civil registration and vital statistics, disease registration, health service use, air pollution monitoring, satellite imaging, disease notifications and other health-related data. GBD 2019 quantifies health loss, including 369 diseases and injuries, and for 87 risk factors in 204 countries and territories around the world, which the data were assessed using spatiotemporal Gaussian process regression, DisMOd-MR 2.1, a Bayesian meta-regression method, or alternative methods for age-sex-location-year exposure (18, 19). These methods have been introduced before and the data and results are available from GBD Results Tool GHDx (healthdata.org) (December 16, 2021).

### Case Definition and Data Sources

GBD 2019 has a risk hierarchy, using CRA to assess the disease burden of risk factors, in which LBMD is defined as a level 3 risk factor, whose exposure is defined as standardized mean bone mineral density values measured by dual X-ray absorptiometry at the femoral neck in g/cm². The theoretical minimum risk exposure level is determined based on 99th percentile of NHANES 1988-2014 by age and sex (23).

In healthy adults’ population, bone mineral density (BMD) appears to be approximately gaussian normal distribution, therefore an individual’s BMD can be valued in standard deviation (SD) units in relation to the reference population. For women, according to WHO and the International Osteoporosis Foundation, low bone mass (osteopenia) is defined as the value for BMD more than 1.0 but less than 2.5 SD below the young adult female reference mean (T-score less than -1 and greater than -2.5 SD) and osteoporosis is the value for BMD 2.5 or more SD below the young adult female reference mean (T-score less than or equal to -2.5 SD). Osteoporosis is also diagnosed based on presence of fragility fractures in the absence of other metabolic bone disorders and even with a normal bone mineral density (T-score) (24–26). International Classification of Diseases (ICD) code list that maps to the global burden of disease cause of death, falls is defined as ICD10 is W00-W19.9 and ICD9 is E880-E886, and E888, as the third level coding strategy in GBD, is one of the unintentional injuries.

The data we are interested in this study consists of: a) global data of LBMD and LBMD-related falls on deaths and DALYs as absolute numbers and age-standardized rates (per 100 000 population) for all age groups; males, females, and both sexes combined; and 204 countries and territories annually from 1990 to 2019. b) Global prevalence, deaths and DALYs of falls as absolute numbers and age-standardized rates (per 100 000 population) by gender and age groups from 1990 to 2019.

Ethical approval and informed consent were not required for this study, as GBD 2019 used DE-idented vetted and approved by the Institutional Review Board of the University of Washington, aggregated data, exempted informed consent, and did not risk disclose personal identity. Used to estimate LBMD and falls, more detailed information of the original data source, see GBD2019 data input source tools website (http://ghdx.healthdata.org/gbd2019/data-input-sources).

### Disease Burden of LBMD Risk and Fracture

We used DALYs and mortality to estimate the global burden of LBMD. DALYs is a pooled indicator of population health, which measures the health status of a population. The goal is to give individuals a standard life expectancy in full health, which has two aspects: years of life lost (YLLs) and years lived with disability (YLDs). YLLs was used to show the burden of premature death from LBMD and YLDs was used to reflect disability-weighted years of life with long-term or short-term health loss. YLDs was calculated by the prevalence of different disease sequelae and injury sequelae multiplied by disability.

Attributing the health burden to osteoporotic fractures was searched by setting LBMD as a risk factor and falls as a cause of injury in GBD 2019. This database used available hospital data to estimate the proportion of injury deaths during admission that could be ascribed to fractures (18). Previous studies that looked at data from hospitals in Brazil, Canada, Mexico and the United States found that falls accounted for a large proportion of deaths, especially among the elderly, and hip fractures were the main cause of death. GBD 2019 restricted assessment of the health burden of LBMD to a list of causes that were deemed to cause fractures: falls, pedestrian road injuries, and other violence injuries. Most high-energy injuries can directly result in fractures because of injuries and violence, not LBMD. Therefore, based on the above research and the research purpose of this paper, most osteoporotic fractures limited to falls are expected to be coded (20). LBMD-related falls can be considered as LBMD-related osteoporotic fractures.

### Socio-Demographic Index

The socio-demographic index (SDI) is a composite indicator of the social background and economic conditions that affect health in each country and region. It is a geometric mean of 0 to 1 and includes per capita income, the average educational level of the population aged 15 or above, and the fertility rate of women under 25. The GBD 2019 and World Bank standards are divided into five parts, high SDI (> 0.81), high-middle SDI (0.70-0.81), middle SDI (0.61-0.69), low-middle SDI (0.46-0.60), and low SDI (< 0.46).

### Statistical Analysis

Data is represented by values with a 95% uncertainty interval (UI). Age-standardized mortality rates and years of life are expressed as figures per 100 000 population. Most statistical analyses were performed using SPSS 24.0(Statistical Product and Service Solutions) software unless otherwise specified, and Prism Version 9 (GraphPad, San Diego, California) was used for all images. Pearson correlation analysis and curve fitting method were used to analyze the relationship between SDI and disease burden. A P value less than 0.05 was considered statistically significant.

## Results

### Global Trends of DALYs and Mortality Attributable to LBMD by Years

**Figure 1:**
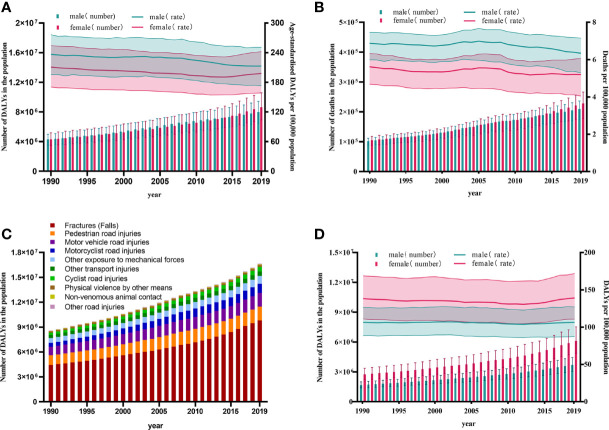
Trend of LBMD and its related fractures at the global level from 1990 to 2019. **(A)** Trends in numbers and age-standardised rates of DALYs of LBMD at the global level,1990-2019; **(B)** Trends in numbers and age-standardised rates of deaths of LBMD at the global level, 1990-2019; **(C)** Composition of different causes at the risk of LBMD by DALYs at the global level, 1990-2019; **(D)** Trends in numbers and age-standardized rates of DALYs of fractures at LBMD risk at the global level, 1990-2019.DALYs, Disability-adjusted life years; LBMD, Low bone mineral Density; Error bars indicate the 95% uncertainty interval (UI) for numbers and rates.

In generally, the global burden of disease attributable to LBMD from 1990 to 2019 increased dramatically, with elevating in the total number of deaths and DALYs (**Figure 1A**). The global DALYs number contributable to LBMD doubled from 8.6 million (95% UI: 10.14-7.04) in 1990 to 16.6 million (95% UI: 20.04-13.50) in 2019. The risk of LBMD was higher in females than in males when it comes to gender. In 2019, compared with LBMD contributed to 4297319 (95% UI: 5 182 046-3 478 535) DALYs in female whereas 4 291 617 (95% UI: 4 989 401-3 521 616) for male in 1990, LBMD doubled in female 8 656 587 (95% UI: 10 586 101-6 935 384) and 7 990 880 (95% UI: 9 429 640-6 480 003) in male (**Figure 1A**). As in 2019, the number of deaths due to LBMD risk increased to 209 586 (95% UI: 236 460-173 630) among male and 228 298 (95% UI: 266 439-177 697) among female. Contrast with 1990, there was an increase of 111.16%, in global death toll (**Figure 1B**).

After the data were standardized for age, the DALYs rate showed a slight downtrend from 226.57 (95% UI: 268.08-185.26) in 1990 to 206.85 (95% UI: 248.69-167.92) per 100 000 population in 2019, as well as, the mortality rate showed a similarly trend from 6.26 (95% UI: 6.88-5.36) in1990 to 5.74 (95% UI: 6.51-4.72) per 100,000 population in 2019 (**Figures 1A, B**).

### Disease Burden of Fractures Due to LBMD by Years

LBMD was a risk factor for many injuries. According to DALYs, fractures accounted for the highest proportion (58.9%) of all injuries related to LBMD worldwide in 2019, followed by pedestrian road injuries (9.99%), motor vehicle road injuries (9.81%), motorcyclist road injuries (6.74%), other exposure to mechanical forces (5.59%), other transport injuries (3.28%), cyclist road injuries (3.26%), physical violence by other means (1.35%), non-venomous animal contact (0.53%) and other road injuries (0.52%) (**Figure 1C**).

The absolute values of deaths and DALYs were growing year by year as a result of fractures with LMBD. Among males, DALYs increased from 1 678 544 (95% UI:2 001 436-1 392 292) in 1990 to 3 704 444 (95% UI:4 440 007-3 031 796) in 2019, while females climbed by 2.21 times from 2 758 245 (95% UI:3 361 499-2 217 636) to 6104020 (95% UI:7 540 557-4 860 689). In addition, the death toll has risen. The number of deaths of fractures caused by LBMD reached 301,482 (95% UI:345 110 -240 323) worldwide in 2019, 2.49 times that in 1990: Males accounted for 39.8% of all deaths, despite female deaths decreasing marginally from 62.5% to 60.2%, to 181,635 (95% UI:213 852-136 974), still more than 1.5 times that of males (**Figure 1D**).

### Differences of Disease Burden in LBMD and Fractures by Gender

**Figure 2:**
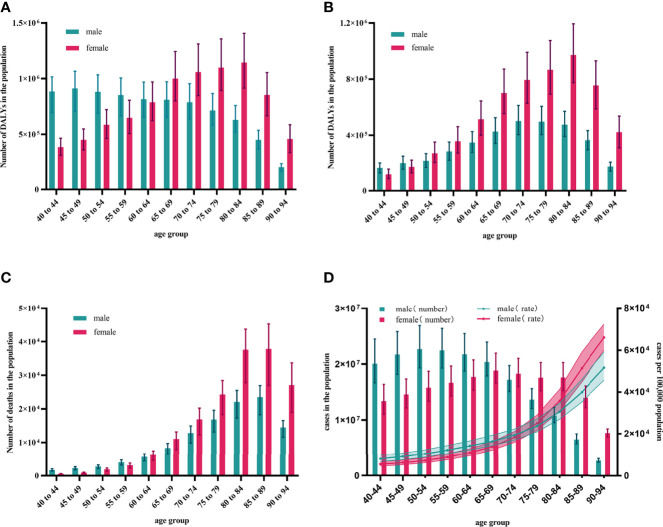
Global burden of LBMD and its related fractures by age groups in 2019. **(A)** DALYs number of LBMD at the global level, 2019; **(B)** DALYs number of fractures at LBMD risk at the global level, 2019; **(C)** Deaths number of fractures at LBMD risk at the global level, 2019; **(D)** Age-specific numbers and rates of fractures prevalent cases by gender, 2019. DALYs, Disability-adjusted life years; LBMD, Low bone mineral density; Error bars indicate the 95% uncertainty interval (UI) for numbers.

LBMD led to a rapidly increase in DALYs and deaths in female from 4 297 319 (95% UI: 5 182 046-3 478 535) and 105 267 (95% UI: 117 964-88 278) in 1990 to 8 656 587 (95% UI: 10 586 101-6 935 384) and 228 297.905 (95% UI: 266 439-177 697) in 2019, with an increase of 101.44% and 116.87%. As for male, the DALYs and deaths improved from 4 291 617 (95% UI: 4 989 401-3 521 616) and 102 100 (95% UI: 111 585-87 870) in 1990 to 7 990 880 (95% UI: 9 429 640-6 480 003) and 209 586 (95% UI: 236 460-173 630) in 2019, with an increase of 86.20% and 105.28%. Female, on average, has a higher risk of LBMD than male, and this gender disparity was projected to widen in the future. Deaths of LBMD-related fractures has increased dramatically in both male and female over the last few decades, reaching 119 846 (95% UI:136 010-98 206) for male and 181 635 (95% UI:213 8512-136 6974) for female in 2019. Impressively, from 1990 to 2019, female deaths rates were higher than male, and the discrepancy appeared to have widened over time. Furthermore, LBMD-related fractures in DALYs and deaths was primarily found in adults ≥40 years old. Males in the 85-89 age had the greatest deaths burden in 2019, accounting for 20.58% of all male deaths (95% UI:26 948-18 268), and the 80-89 age group accounted for nearly half of all male deaths. As for female, deaths number of LBMD-related fractures was 37 790 (95% UI:45 229-26 976), 1.60 times as many as males at the age of 85-89. The gender divide among those over 40 years old widened as they became older: deaths of female was 0.31-fold those of males at the age of 40-44, a shocking 1.70-fold between 80-84 years old, and 1.874-fold between 90-94 years old. Female had a higher health burden than male, which was reflected not only in the number of fatalities, but also in DALYs. Both male and female had a unimodal trend in DALYs. Male aged 70-74 years had the highest DALYs: 50 1500 (95% UI: 612 276-404 681), with 70-89 years group accounting for 50.36% of total burden of male. Between the age of 80-84, the peak number of females was 971 411 (95% UI:1 193 500-774 109). Female growth rates were higher than male beyond the age of 40. Female aged 40-44 have 0.73 times the disease burden of males, while female aged 90-94 have 2.43 times that of male (**Figure 2**).

### The Burden of Fractures Due to LBMD by Countries and Regions

**Figure 3:**
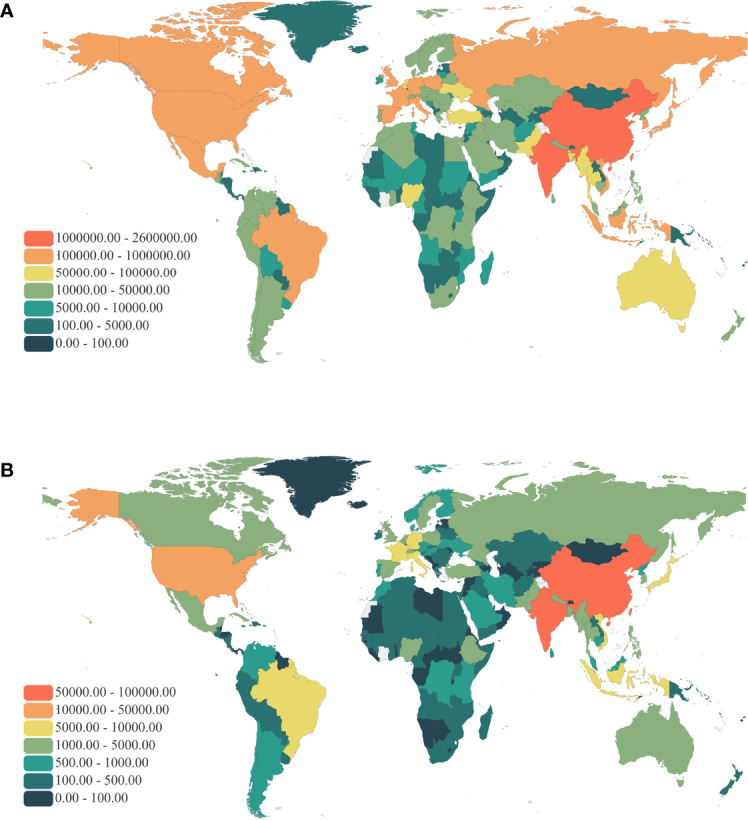
Global map of health burden of LBMD related fractures in 2019. **(A)** DALYs number; **(B)** Death number. DALYs, Disability-adjusted life years; LBMD, Low bone mineral density.

The study found that the burden of LBMD-related fractures was greater in developed countries and in developing countries with larger populations. In the GBD 2019 study, data from 204 countries and territories were analyzed. The five countries with the highest DALYs number in fractures due to LBMD in 2019 were India 2 510 288 (95% UI:2 971 348 -2 072 778); China 1 839 375 (95% UI:2 316 329-1 346 044); United States of America 819 445 (95% UI:1 041 431-644 729); Japan 323 094 (95% UI:419 012-248 280); Germany 297 944 (95% UI:380 978-228 142). They, in turn, accounted for 25.59%, 18.75%, 8.35%, 3.29%, and 3.04% of the global total LBMD-related fractures burden. The countries with the highest number of deaths due to LBMD-related fractures in 2019, in order, India 93 675 (95% UI:110 965-74 053), China 56 639 (95% UI:71 875-30 514), United States of America 22 174 (95% UI:24 927-18 183), France 9321 (95% UI:11 214-6840), Germany 8542 (95% UI:9892-6815), with the proportion of 31.07%,18.79%,7.36%, 3.09%,2.83% (**Figure 3**).

### The Association Between SDI and Fractures Due to LBMD

**Figure 4:**
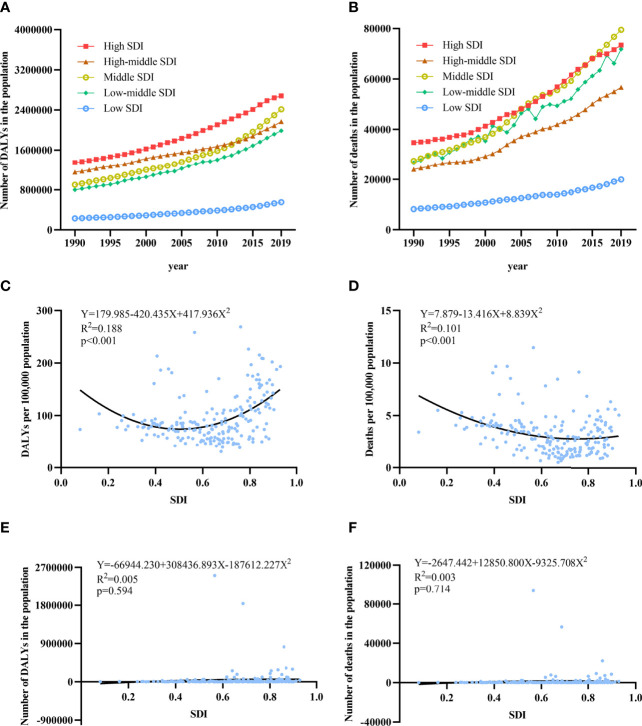
The association between SDI and burden of LBMD related fractures. **(A)** Trends in DALYS number of fractures at LBMD by SDI quintiles in both sexes,1990-2019; **(B)** Trends in DALYS rate of fractures at LBMD by SDI quintiles in both sexes,1990-2019; **(C)** Association between DALYs rate and SDI in both sex in 2019; **(D)** Association between deaths rate and SDI in both sex in 2019; **(E)** Association between DALYs number and SDI in both sex in 2019; **(F)** Association between deaths number and SDI in both sex in 2019; SDI, Socio-demographic Index.

According to the SDI classification, the 204 countries and territories was divided into 33 low, 40 low-middle, 44 middle, 44 high-middle and 43 high SDI countries or territories. We found that the three countries with the highest DALYs and deaths were India, China and the United States. The number of deaths and DALYs in all three countries is increasing year by year. The combined DALYs and deaths rate of male and female were positively correlated with SDI (linear regression) in all countries and regions: DALYs rate was 179.985-420.435SDI+417.936SDI^2^(R^2^ = 0.188, p<0.001); Deaths rate was also positively correlated with SDI, deaths rate 7.879-13.416SDI+8.839 SDI^2^(R^2^ = 0.101, p<0.001). And DALYs number was -66 944.230 + 308 436.893SDI-187 612.227 SDI^2^ (R^2^ = 0.005, p=0.594); Deaths number was -2647.442+12 850.800SDI-9325.708SDI^2^ (R^2^ = 0.003, p=0.714) (**Figures 4C–F**).

For countries and regions with high SDI, the number of DALYs increased from 1 348 472 (95% UI:1 699 379-1052555) in 1990 to 2 681 727 (95% UI:3 409 105-2 086 644) in 2019, an increase of 98.87%. The number of countries and regions in the high-middle SDI increased from 1 153 070 (95% UI:1 431 916-917 557) to 2 168 703 (95% UI:2 739 295-1 706 491), an increase of 88.08%. The Middle SDI increased 167.96% from 901 213 (95% UI:105 619-747 672) to 2 414 849 (95% UI:2 929 129-1 905 732). The number of low-middle SDI countries and territories reached 1 985 931 (95% UI:2 340 410-1 649 307) in 2019, 2.64 times that of 1990. The DALYs with low SDI countries and territories also reached 552 813 (95% UI:642 702-467 246) in 2019, 2.40 times that of 1990. In female, the increase of fractures associated with LBMD was significantly higher in low-middle and middle SDI countries than in male. And in middle SDI countries and territories, female DALYs number increased by 1.74 times from 529 735 (95% UI:627 607-427 067) in 1990 to 1 449 610 (95% UI:1 768 625-1 132 004) in 2019. In low-middle SDI, an increase of 1.62 times (**Figures 4A, B**). The upward trend of males in different SDI countries was the same as that of females, and the fastest upward trend was mainly in low-middle and middle SDI countries and territories.

## Discussions

Based on an in-depth analysis of the data from GBD study 2019, this study estimates the global burden of LBMD and LBMD-related fractures. From 1990 to 2019, the total number of deaths and DALYs from LBMD and LBMD-related fractures increased significantly, consistent with the rising trend in the number of patients and deaths from osteoporosis (27). After age was standardized to adjust for population and age structure, LBMD-related fractures death rate and DALYs rate were slowly declining. The changes in rates showed that the death of absolute number rose is largely due to population growth, moreover also increasing the number of cases related with the incidence of falls. This study showed that LBMD is responsible for half of the fall in recent years. Fragility fractures due to osteoporosis caused a significant and growing economic burden on healthcare systems and societies worldwide, so IOF (International Osteoporosis Foundation) calls for priority to be given to prevention to support the effective management of fragility fractures, thereby avoiding the escalation of pain and suffering and associated costs.

The results of the analysis by age showed that there was a unimodal distribution of deaths of LBMD-related fractures both in male and female. In all age groups between 40 to 59 year, LBMD-related fractures caused more deaths in male than in female, while in the age groups 60 year and above, the result was reversed. The higher mortality of male in early life may be related to their social work. In the current social division of labor, male have more physical labor, and the probability of violent injury is higher than female (28). DALYs surpassed male as early as age 50. Our findings are consistent with most previous researches (9, 11). The result was Partly because of differences in bone mineral density between males and females at maturity, and also because of the rate of cortical bone loss in males and females: the bone mass of the human body reached the peak in early adulthood, and then from roughly the fifth decade began to decline with the growth of the age, females have begun to have a large number of cortical bone loss in middle age, and males began at 75-year-old. Besides, females in a variety of factors influence, had an acceleration period of bone loss in the menopausal transition, with an annual rate of bone loss of 1-2% (29). Before menopause, estrogen played a protective role against women, and in older women. Reduction in bone mass was associated with low level of biological activity sex steroids and higher levels of follicle stimulating hormone and bone turnover markers. Similarly, as the growth of the age, male and female fracture frequency was increasing, a combination of reduced bone density and an increased tendency to falls (30). In addition, females generally lived longer than males, so they may be exposed to low bone mineral levels for longer periods of time than males, and 61% of osteoporosis fractures occurred in females, for a ratio of 1.6 to 1. And studies have shown that females are twice as likely as males to have sustained fractures (7).

Based on the disease burden of LBMD related fractures, we found that countries with high DALYs rates were mostly located in Western Europe, Northern Europe and The Indian Peninsula after standardizing the data according to population and age. Geographic and ethnic differences may be associated with the distribution of osteoporotic fractures. The incidence of osteoporotic fractures due to LBMD may be ethnically related, with racial differences in fragility fractures being greater than in any other fracture. Black people have the lowest fracture rates for both male and female, so white female are 4.7 times more likely to have a fragility fracture than black female, and white male are 2.7 times more likely than black male. South Asian male also had higher fracture rates than black and mixed-race male (31). In general, people who live further away from the equator might have higher rates of fractures (32). These might be partly responsible for the high osteoporotic fracture burden in places such as Northern Europe. Other studies have found that incidence of fractures in whites are higher than in blacks and Asians over the age of 50, but the rates in blacks, Asians and Hispanics gradually surpass those in whites as people age (33). This might explain the higher fracture burden associated with LBMD in South Asia, particularly India, in recent years.

We found that countries or territories with a higher burden of fractures associated with LBMD were found in both economically advanced and less developed regions. This suggested that osteoporotic fractures were not only related to gene, but also economic situation. We found that from 1990 to 2019, countries and regions with high SDI had the highest DALYS number, especially in the United States and The European Union. After standardizing the population structure of the data, the increase rate of osteoporotic fractures has decreased in countries and regions with high SDI in recent years. On the contrary, countries and regions with middle and low-middle SDI levels showed a trend of rapid rise, and even in recent five years, DALYs rate exceeded that of countries and regions with high SDI, among which India was the most outstanding. The higher burden of disease in regions such as developed countries might be related to their current large populations, and the recent decline in the rate of osteoporotic fractures coincide with the approval of bisphosphonates for the treatment of osteoporosis. The use of anti-osteoporosis drugs has been observed to reduce the incidence of hip fractures in Belgium, the United States, Denmark and elsewhere (34, 35). Combined estrogen-progestin replacement therapy was associated with significant reduction in hip fracture was also proved in the Women’s Health Initiative, whereas the announcement of the Women’s Health Initiative (WHI) in 2002 showed that hormone replacement therapy (HRT) had more detrimental than beneficial effects (36, 37). Since then, despite HRT remains the most effective treatment for vasomotor symptoms (VMS) and the genitourinary syndrome of menopause, the popularity and use of hormone therapy declined (38, 39). However, BMS (British Medical Society), IMS (International Menopause Society) and NAMS (The North American Menopause Society) have confirmed the benefits of HRT in the treatment of osteoporosis, arguing that the conclusion of the 2002 WHI study was biased. They claimed that for women aged younger than 60 years or who are within 10 years of menopause onset and have no contraindications, estrogen-based treatments still have a major role in the treatment and risks can be minimized and benefits maximized with selection of individualized circumstances (40, 41). With the development of modern medicine, a number of drugs such as selective estrogen receptor modulators, bisphosphonates, denosumab, have been developed for the treatment of osteoporosis fractures. The use of these drugs may further reduce the incidence of osteoporotic fractures (2).

In this study, there was a U-shaped relationship between SDI and DALYs rate, so did SDI and mortality. SDI is a great indicator in the assessment of social development and the relation between SDI and LMBD-related fractures was influenced by multiple factors. The occurrence of osteoporotic fractures is affected by the interaction of various factors such as exercise and nutrition. Lower socioeconomic status means heavier lifting and more bending motion which can lead to vertebral fractures and contingencies. On the contrary, unhealthy lifestyle habits increase as the result of economic status development, and exercise loses its protective effect on bone density (42). Despite exercise, nutrition condition can also affect osteoporotic fractures. There is a correlation between average body weight and the incidence of fractures, with both underweight and overweight increasing the incidence of fractures, especially in people with a body mass index <20 kg/m^2^ (43). Furthermore, medical conditions and life expectancy are two other reasons explaining the relationship. Due to the poor medical care and lack of health education, timely treatment cannot be obtained lead to higher risk of the disability and mortality in people with fractures in countries with low level of social development. Although the death rate from osteoporotic fractures has declined due to improvements in medical care and life expectancy, the duration of disability and loss of quality of life is more pronounced, which explains why osteoporosis is a heavier problem in developed countries (44, 45). Therefore, the prevention and treatment of osteoporotic fracture must be emphasized in the transition of economic and social development.

Osteoporosis, as a chronic disease, was closely related to the aging of the population. Previous researches showed that the number of men and women aged 65 and over in Europe wound increase by 50.6 percent over the next 25 years. For developing countries, where the burden of disease wound be more pronounced in the future and the total population and life expectancy of the elderly will more than triple in the next 25 years. The higher DALYs and death rates reflected potential modifiable factors that wound be more significant. As the burden of disease increases, so will the economic burden of osteoporotic fractures, both directly and indirectly. With the cost of osteoporotic fractures rising faster than the general rate of inflation in almost every country worldwide (3), osteoporotic fractures will have greater significance for healthcare planning. Paying more attention to the prevention and care of osteoporotic fractures will bring more benefits and reduce social and medical pressure.

Genetic factors were important in the development of osteoporotic fractures, but differences between ethnic groups can be modified by reversing lifestyle. Some lifestyles such as low milk intake, smoking, lack of sunlight exposure, lower BMI, and physical activity (46), can cause the incidence of hip fracture to rise further. The high prevalence of unhealthy lifestyle habits such as smoking and alcohol abuse in lower socioeconomic levels, coupled with more pronounced poor diet structure and quality, has been shown to have adverse effects on bones (1, 47). Therefore, the most logical and cost-effective prevention strategy is to encourage the population to quit smoking and avoid excessive alcohol use. And provide advice on consuming adequate calcium and vitamin D, as well as medical advice (48). Of course, inadequate understanding of osteoporosis in developing countries may also play a role, with knowledge of osteoporosis and its risk factors currently low in the Indian cohort of men and women. There is a need to create awareness programmers for both female and male, especially for those with lower education, lower socio-economic status and a history of osteoporosis.

This study also has some limitations. The current disease burden of osteoporotic fractures may be underestimated for several reasons. Firstly, osteoporosis is likely to be missed as a potential cause of death because osteoporotic fractures and death has long time interval (49). Secondly, DXA screening for diagnosis and monitoring of osteoporosis is generally reserved for high-risk patients currently. Compared to other chronic diseases such as hypertension, obesity, and diabetes mellitus, bone mineral density test is relatively expensive and need higher technical requirement, therefore relevant data source is limited. The parameters of DXA devices in different countries are divergent, leading to some changes in the values. Besides, there are many diagnostic criteria for osteoporosis, such as the international standard recommended by WHO, NOF (National Osteoporosis Foundation) and ISCD (International Society for Clinical Densitometry) (50–52). There are information gaps in different health information systems in different countries and regions. In addition, the deficiency of this study also include that the disease burden associated with LBMD in GBD 2019 is restricted to hip fractures. However, for patients with osteoporotic fractures, there are also lumbar vertebrae, thoracic compression fractures, and upper limb fractures, etc. Besides, population with the same LBMD has different fracture rates at different risks. Due to limited data, it was not possible to quantify bone mineral density, and other information was lacking on subjects’ history of fracture, bone metabolic diseases, or treatments that might affect bone metabolism (53). We recommend that future studies include multiple osteoporotic fractures, such as vertebrae and radius fractures, and quantify individual risk factors to provide as comprehensive patient information as possible.

In conclusion, this study suggested that LBMD and fracture is a growing global health burden. Female had a higher burden of disease than male, and the gap widened with age. Increasing education and dissemination of osteoporosis, improving resource allocation, and paying more attention on screening and treatment of osteoporosis could help reduce the global burden of disease attributable by LBMD and fracture, especially in low-middle and middle SDI countries and territories.

## Data Availability Statement

The original contributions presented in the study are included in the article/**Supplementary Material**. Further inquiries can be directed to the corresponding authors.

## Author Contributions

YS and XH conceived the study, collected data, performed the statistical analysis and participated in writing and preparation of the report. JW, XL, XZ and ZZ were involved in the data collection, interpretation of the data and preparation of the report. XP, JX, and JQ analyzed the data and revised the report. TZ, and LY revised the report. P-FS, YR, and HJ designed and coordinated the study, acquired funding, performed the statistical analysis and participated in writing and editing the final report. P-FS assumes full responsibility for the overall content of this report. All authors contributed to the article and approved the submitted version.

## Funding

This work was supported by grants from the National Natural Science Foundation of China [grant numbers 81870564 to P-FS].

## Conflict of Interest

The authors declare that the research was conducted in the absence of any commercial or financial relationships that could be construed as a potential conflict of interest.

## Publisher’s Note

All claims expressed in this article are solely those of the authors and do not necessarily represent those of their affiliated organizations, or those of the publisher, the editors and the reviewers. Any product that may be evaluated in this article, or claim that may be made by its manufacturer, is not guaranteed or endorsed by the publisher.
