# Supplementary material for: The Global Burden of Osteoporosis, Low Bone Mass, and Its Related Fracture in 204 Countries and Territories, 1990-2019
Source: Front Endocrinol (Lausanne). 2022 May 20;13:882241. doi: 10.3389/fendo.2022.882241 (PMC9165055; doi:10.3389/fendo.2022.882241)
Supplement: Supplementary file 1 [file DataSheet_1.docx]

Supplementary Material

# Supplementary Figures and Tables

## Supplementary Figures


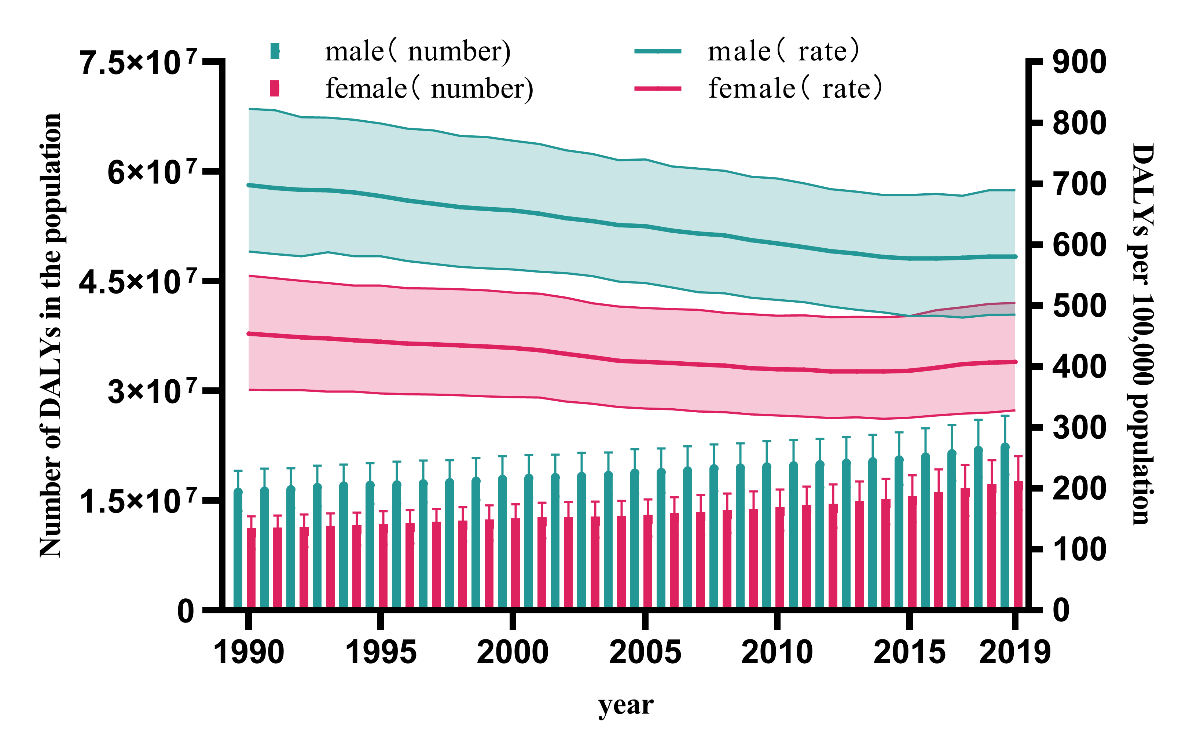


**Supplementary Figure 1.** Trends in numbers and age-standardised rates of DALYs (Disability-adjusted life years.)of fractures (falls) at the global level,1990-2019. Error bars indicate the 95% uncertainty interval (UI) for numbers; Shading indicates the 95% UI for rates.


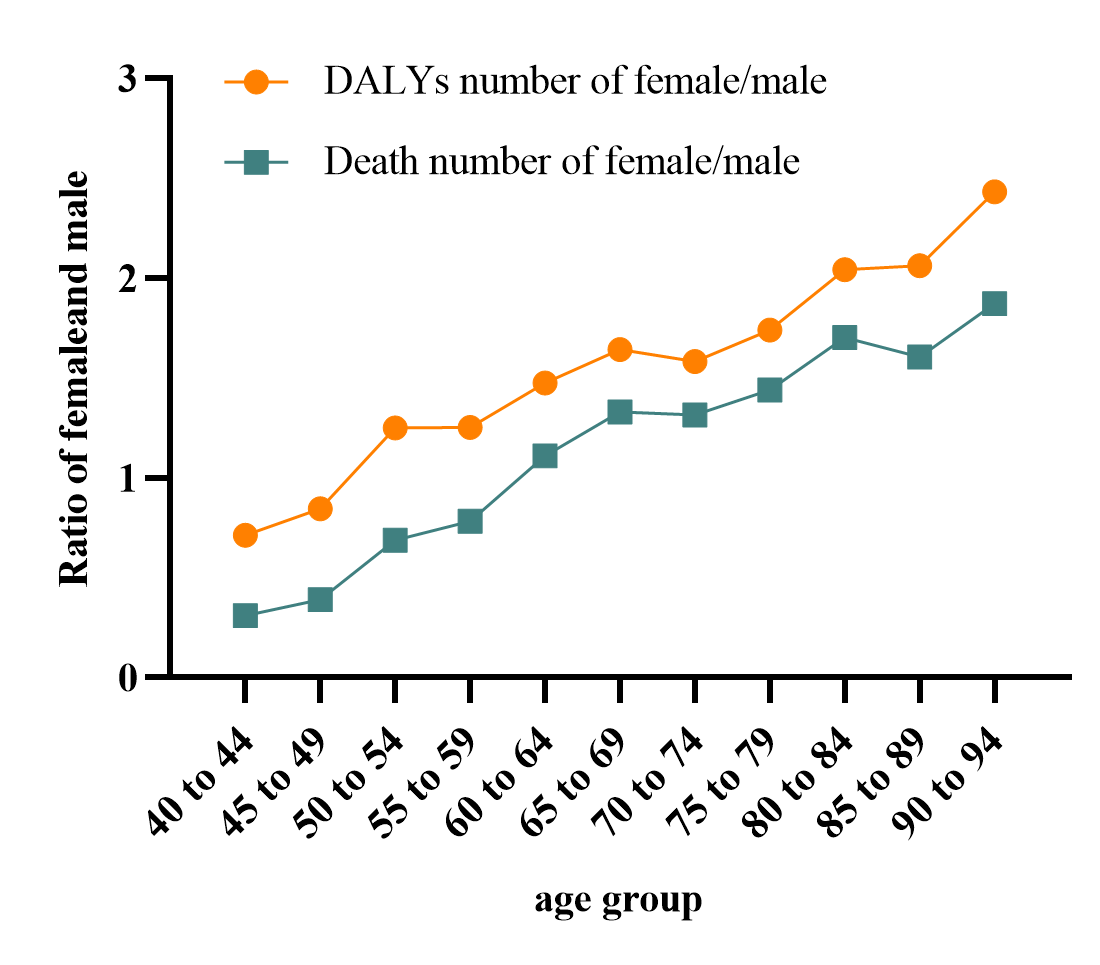


**Supplementary Figure 2** The ratio of female and male with DALYs and deaths number of LBMD related fractures (falls) in different age groups, in 2019. DALYS, Disability-adjusted life years; LBMD, Low bone mineral density.


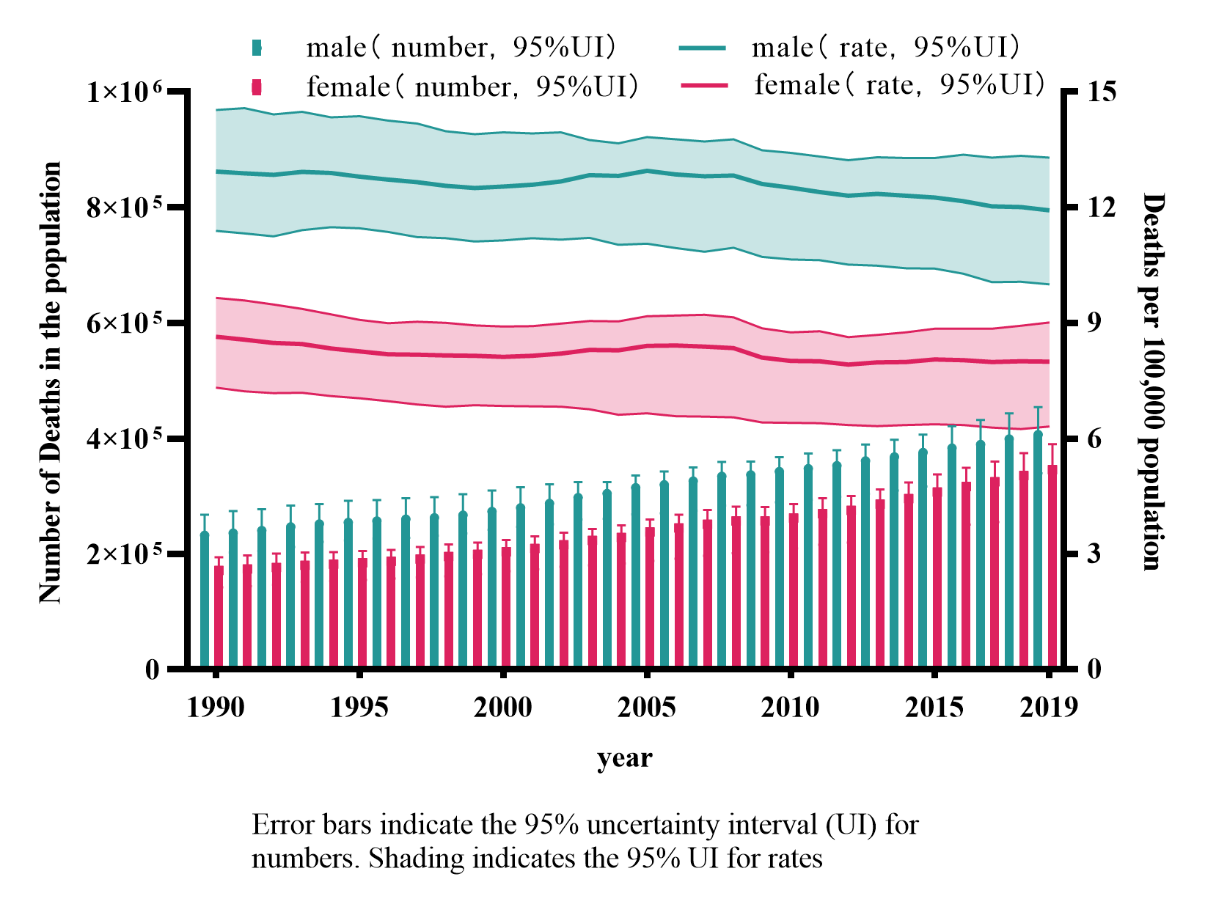


**Supplementary Figure 3** Trends in numbers and age-standardised rates of deaths of fractures at the global level,1990-2019. Error bars indicate the 95% uncertainty interval (UI) for numbers; Shading indicates the 95% UI for rates.


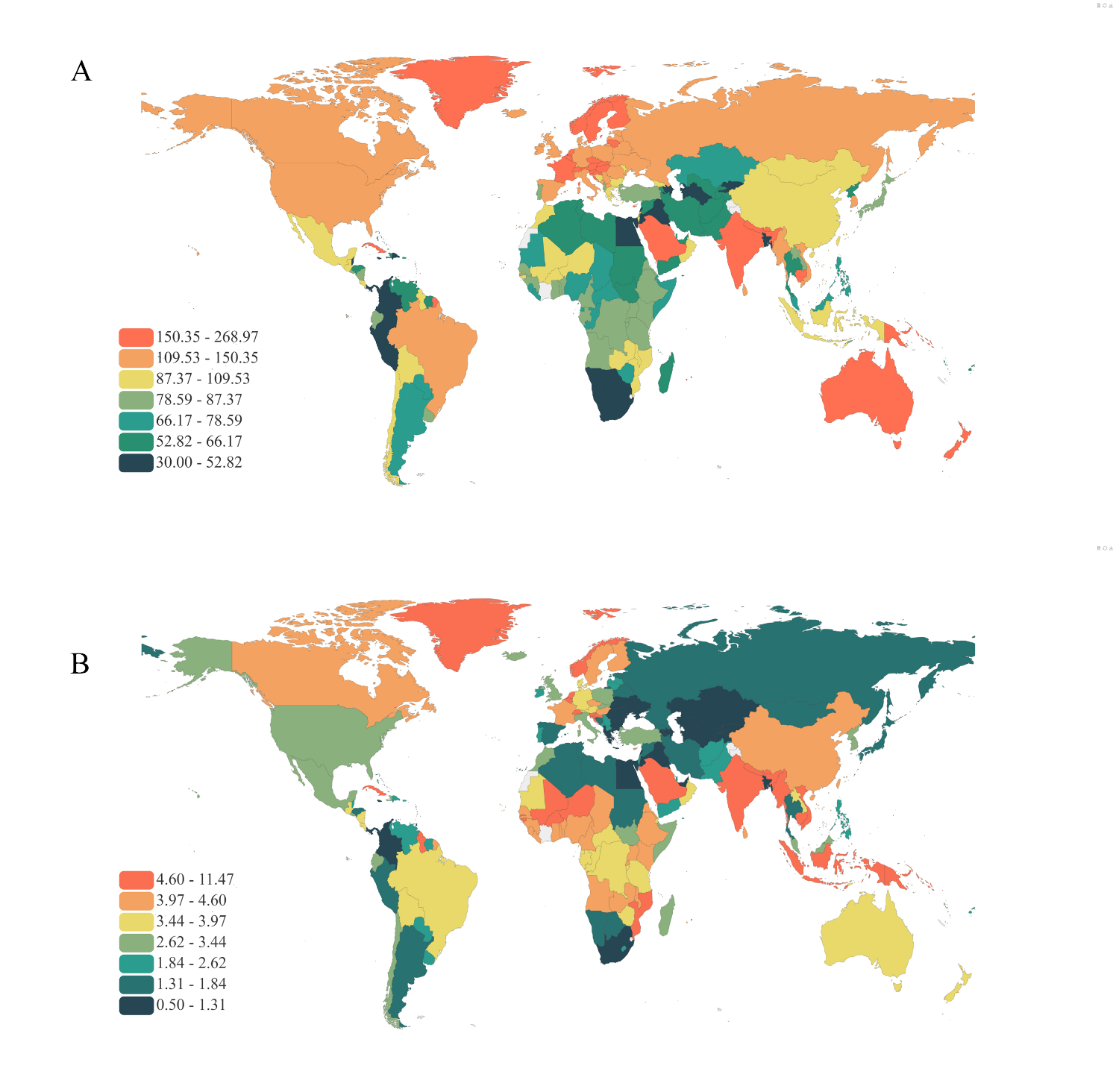


**Supplementary Figure 4** Global map of health burden of LBMD related fractures in 2019. (A) DALYs rate; (B) Death rate. DALYS: Disability-adjusted life years; LBMD: Low bone mineral density;

**1.2 Supplementary Table**

Table S1. Numbers and age-standardized rates of YLLs and YLDs of LBMD at the global level by sex, 1990-2019.

| **year** | **sex** | **YLLs number** | **upper** | **lower** | **YLLs rate** | **upper** | **lower** | **YLDs number** | **upper** | **lower** | **YLDs rate** | **upper** | **lower** |
| --- | --- | --- | --- | --- | --- | --- | --- | --- | --- | --- | --- | --- | --- |
| 1990 | both | 4506871 | 4918270 | 3862879 | 117 | 128 | 101 | 4082065 | 5511964 | 2923343 | 109 | 147 | 78 |
|  | male | 2617764 | 2912521 | 2197431 | 141 | 156 | 120 | 1673853 | 2254961 | 1185298 | 95 | 128 | 67 |
|  | female | 1889107 | 2102351 | 1593342 | 93 | 103 | 79 | 2408212 | 3243837 | 1732145 | 118 | 159 | 85 |
| 1991 | both | 4595134 | 5003993 | 3935786 | 117 | 127 | 100 | 4151903 | 5607297 | 2972982 | 108 | 146 | 78 |
|  | male | 2672928 | 2957378 | 2255258 | 141 | 155 | 120 | 1701497 | 2291573 | 1205353 | 94 | 127 | 67 |
|  | female | 1922206 | 2149327 | 1623300 | 92 | 103 | 78 | 2450407 | 3299149 | 1762884 | 117 | 158 | 85 |
| 1992 | both | 4693532 | 5109195 | 4005342 | 116 | 126 | 99 | 4223877 | 5705197 | 3025616 | 108 | 145 | 77 |
|  | male | 2730809 | 3022484 | 2307484 | 140 | 154 | 119 | 1731014 | 2331819 | 1225492 | 94 | 126 | 66 |
|  | female | 1962723 | 2182605 | 1657249 | 92 | 102 | 77 | 2492863 | 3355790 | 1792848 | 117 | 157 | 84 |
| 1993 | both | 4840523 | 5250775 | 4142612 | 117 | 127 | 100 | 4297850 | 5802569 | 3074997 | 107 | 144 | 77 |
|  | male | 2826110 | 3118399 | 2388081 | 141 | 155 | 121 | 1761674 | 2373595 | 1247685 | 93 | 126 | 66 |
|  | female | 2014413 | 2238031 | 1709520 | 92 | 102 | 78 | 2536176 | 3412941 | 1821739 | 116 | 156 | 83 |
| 1994 | both | 4955308 | 5374016 | 4229080 | 116 | 126 | 100 | 4378077 | 5910909 | 3132474 | 107 | 144 | 76 |
|  | male | 2899849 | 3187579 | 2451649 | 141 | 155 | 120 | 1795040 | 2421338 | 1269981 | 93 | 125 | 65 |
|  | female | 2055458 | 2279268 | 1750160 | 92 | 102 | 78 | 2583037 | 3474144 | 1854407 | 116 | 155 | 83 |
| 1995 | both | 5027235 | 5460689 | 4293310 | 115 | 125 | 98 | 4466835 | 6033900 | 3196156 | 106 | 143 | 76 |
|  | male | 2948134 | 3231819 | 2487956 | 140 | 153 | 120 | 1832177 | 2470876 | 1294869 | 93 | 125 | 65 |
|  | female | 2079101 | 2297919 | 1771029 | 90 | 100 | 77 | 2634657 | 3541448 | 1892862 | 115 | 155 | 83 |
| 1996 | both | 5107463 | 5535526 | 4377957 | 114 | 124 | 98 | 4569067 | 6168871 | 3264538 | 106 | 143 | 76 |
|  | male | 2995003 | 3269659 | 2507159 | 139 | 151 | 118 | 1877268 | 2532501 | 1327603 | 93 | 125 | 65 |
|  | female | 2112460 | 2330215 | 1789233 | 90 | 99 | 76 | 2691799 | 3617974 | 1930929 | 115 | 154 | 83 |
| 1997 | both | 5212156 | 5658430 | 4432375 | 114 | 124 | 97 | 4684174 | 6320081 | 3344801 | 106 | 143 | 76 |
|  | male | 3048921 | 3331144 | 2574295 | 138 | 151 | 118 | 1930687 | 2606087 | 1365654 | 93 | 125 | 65 |
|  | female | 2163235 | 2399887 | 1837094 | 90 | 99 | 76 | 2753486 | 3696499 | 1977107 | 115 | 154 | 83 |
| 1998 | both | 5300018 | 5734711 | 4500921 | 113 | 123 | 96 | 4804761 | 6482796 | 3430124 | 106 | 143 | 76 |
|  | male | 3091647 | 3376981 | 2610127 | 137 | 149 | 117 | 1987695 | 2683677 | 1406697 | 93 | 126 | 66 |
|  | female | 2208372 | 2444944 | 1851965 | 89 | 99 | 75 | 2817066 | 3782496 | 2023072 | 115 | 154 | 82 |
| 1999 | both | 5418093 | 5874678 | 4628251 | 113 | 123 | 97 | 4926026 | 6643812 | 3517028 | 106 | 143 | 76 |
|  | male | 3159218 | 3451520 | 2662644 | 137 | 149 | 116 | 2044924 | 2760767 | 1447166 | 94 | 126 | 66 |
|  | female | 2258875 | 2497198 | 1895970 | 89 | 99 | 75 | 2881102 | 3869959 | 2069313 | 114 | 154 | 82 |
| 2000 | both | 5563744 | 6036937 | 4775117 | 113 | 123 | 97 | 5042826 | 6800207 | 3602050 | 106 | 143 | 76 |
|  | male | 3257624 | 3549531 | 2767698 | 137 | 149 | 118 | 2098238 | 2836979 | 1483991 | 94 | 127 | 66 |
|  | female | 2306120 | 2548592 | 1948987 | 89 | 98 | 75 | 2944587 | 3957410 | 2115519 | 114 | 154 | 82 |
| 2001 | both | 5710618 | 6221294 | 4891670 | 113 | 124 | 97 | 5140403 | 6930370 | 3669883 | 106 | 142 | 75 |
|  | male | 3342317 | 3667084 | 2822124 | 138 | 151 | 118 | 2142411 | 2896599 | 1514832 | 94 | 126 | 66 |
|  | female | 2368302 | 2617732 | 2016335 | 89 | 99 | 76 | 2997992 | 4029859 | 2152557 | 113 | 153 | 81 |
| 2002 | both | 5923309 | 6422751 | 5029576 | 114 | 124 | 97 | 5215056 | 7023579 | 3720879 | 104 | 140 | 75 |
|  | male | 3470418 | 3784268 | 2908014 | 139 | 151 | 118 | 2177819 | 2945908 | 1540268 | 93 | 125 | 65 |
|  | female | 2452891 | 2715177 | 2069674 | 90 | 100 | 76 | 3037237 | 4081765 | 2177975 | 112 | 151 | 80 |
| 2003 | both | 6122688 | 6688464 | 5186599 | 115 | 126 | 98 | 5282177 | 7114766 | 3768255 | 103 | 139 | 74 |
|  | male | 3591768 | 3923903 | 3004190 | 140 | 153 | 119 | 2209936 | 2988599 | 1563143 | 92 | 123 | 65 |
|  | female | 2530919 | 2795727 | 2126159 | 90 | 100 | 76 | 3072241 | 4129002 | 2202791 | 110 | 148 | 79 |
| 2004 | both | 6258594 | 6840508 | 5322399 | 115 | 126 | 98 | 5362107 | 7220921 | 3825123 | 102 | 137 | 73 |
|  | male | 3677596 | 4040814 | 3067472 | 140 | 153 | 117 | 2246458 | 3040057 | 1590510 | 91 | 122 | 64 |
|  | female | 2580998 | 2853534 | 2152389 | 90 | 100 | 75 | 3115649 | 4183695 | 2234280 | 109 | 146 | 78 |
| 2005 | both | 6476483 | 7111530 | 5462625 | 116 | 127 | 97 | 5472269 | 7376941 | 3902444 | 101 | 136 | 72 |
|  | male | 3803837 | 4177083 | 3156518 | 141 | 155 | 118 | 2293897 | 3105732 | 1623889 | 90 | 122 | 64 |
|  | female | 2672646 | 2966717 | 2225674 | 91 | 101 | 75 | 3178373 | 4271573 | 2277458 | 108 | 146 | 77 |
| 2006 | both | 6606699 | 7229980 | 5601117 | 115 | 126 | 97 | 5615096 | 7567385 | 4002283 | 101 | 136 | 72 |
|  | male | 3873683 | 4258421 | 3216157 | 139 | 153 | 117 | 2352847 | 3184288 | 1665704 | 90 | 121 | 63 |
|  | female | 2733015 | 3037210 | 2269891 | 90 | 100 | 75 | 3262249 | 4387823 | 2336359 | 108 | 145 | 77 |
| 2007 | both | 6756181 | 7411920 | 5660845 | 114 | 125 | 96 | 5777433 | 7780986 | 4117610 | 101 | 136 | 72 |
|  | male | 3968969 | 4372795 | 3269053 | 139 | 153 | 115 | 2418369 | 3271698 | 1711846 | 90 | 121 | 63 |
|  | female | 2787212 | 3098268 | 2299594 | 89 | 99 | 73 | 3359064 | 4517040 | 2405168 | 108 | 145 | 77 |
| 2008 | both | 6908433 | 7599107 | 5819522 | 113 | 125 | 96 | 5949167 | 8014691 | 4239740 | 101 | 136 | 72 |
|  | male | 4069154 | 4481913 | 3357025 | 139 | 152 | 116 | 2486146 | 3360783 | 1756510 | 90 | 121 | 63 |
|  | female | 2839279 | 3160050 | 2342577 | 88 | 99 | 73 | 3463021 | 4659352 | 2480024 | 108 | 146 | 78 |
| 2009 | both | 6922196 | 7621364 | 5804227 | 111 | 122 | 93 | 6126839 | 8257373 | 4364070 | 101 | 136 | 72 |
|  | male | 4091167 | 4495742 | 3373888 | 136 | 149 | 113 | 2555579 | 3454591 | 1801222 | 90 | 121 | 63 |
|  | female | 2831030 | 3142816 | 2332965 | 86 | 95 | 71 | 3571260 | 4811904 | 2556612 | 109 | 146 | 78 |
| 2010 | both | 7023744 | 7723568 | 5885521 | 109 | 120 | 92 | 6305141 | 8502776 | 4491475 | 101 | 136 | 72 |
|  | male | 4151301 | 4601401 | 3382690 | 135 | 149 | 110 | 2624937 | 3548384 | 1848570 | 90 | 121 | 63 |
|  | female | 2872443 | 3193474 | 2394620 | 85 | 94 | 70 | 3680203 | 4964648 | 2635265 | 109 | 147 | 78 |
| 2011 | both | 7134692 | 7865796 | 5993449 | 108 | 119 | 91 | 6479025 | 8740412 | 4611841 | 101 | 136 | 72 |
|  | male | 4202068 | 4645279 | 3420742 | 133 | 147 | 110 | 2691212 | 3637796 | 1893458 | 90 | 121 | 63 |
|  | female | 2932624 | 3266996 | 2441245 | 84 | 94 | 70 | 3787813 | 5105582 | 2711405 | 109 | 147 | 78 |
| 2012 | both | 7217544 | 7952222 | 6073091 | 107 | 117 | 90 | 6652969 | 8971462 | 4733062 | 101 | 136 | 72 |
|  | male | 4252851 | 4712145 | 3451889 | 131 | 145 | 108 | 2755333 | 3724825 | 1939321 | 89 | 121 | 63 |
|  | female | 2964693 | 3287997 | 2461575 | 83 | 92 | 68 | 3897636 | 5253286 | 2787870 | 109 | 147 | 78 |
| 2013 | both | 7313881 | 8047343 | 6145292 | 105 | 116 | 88 | 6830780 | 9210826 | 4861255 | 101 | 135 | 71 |
|  | male | 4278621 | 4738126 | 3494812 | 129 | 143 | 107 | 2819067 | 3808079 | 1983088 | 89 | 120 | 63 |
|  | female | 3035260 | 3369605 | 2528881 | 82 | 91 | 68 | 4011713 | 5405893 | 2870944 | 109 | 146 | 78 |
| 2014 | both | 7395183 | 8152927 | 6261530 | 104 | 115 | 88 | 7023052 | 9470002 | 4997380 | 101 | 136 | 71 |
|  | male | 4287140 | 4766765 | 3510526 | 127 | 141 | 105 | 2887885 | 3903351 | 2030723 | 89 | 120 | 62 |
|  | female | 3108043 | 3468454 | 2590398 | 82 | 91 | 68 | 4135167 | 5569230 | 2960300 | 109 | 147 | 78 |
| 2015 | both | 7551155 | 8338345 | 6352489 | 103 | 115 | 87 | 7240056 | 9762668 | 5144497 | 101 | 136 | 71 |
|  | male | 4339194 | 4809190 | 3558387 | 126 | 139 | 104 | 2966996 | 4010148 | 2085750 | 89 | 120 | 62 |
|  | female | 3211961 | 3586131 | 2644506 | 82 | 92 | 68 | 4273061 | 5752717 | 3055179 | 109 | 147 | 78 |
| 2016 | both | 7671580 | 8506183 | 6429853 | 103 | 114 | 86 | 7572317 | 10223633 | 5377394 | 102 | 138 | 73 |
|  | male | 4385828 | 4900043 | 3581952 | 124 | 138 | 102 | 3079502 | 4169350 | 2166766 | 90 | 122 | 63 |
|  | female | 3285752 | 3680783 | 2699736 | 82 | 92 | 67 | 4492814 | 6060263 | 3215436 | 112 | 151 | 80 |
| 2017 | both | 7759999 | 8649220 | 6445631 | 101 | 113 | 84 | 7941650 | 10730811 | 5633298 | 104 | 141 | 74 |
|  | male | 4412283 | 4979420 | 3614405 | 122 | 137 | 101 | 3208036 | 4345877 | 2258597 | 91 | 123 | 64 |
|  | female | 3347716 | 3778873 | 2754833 | 81 | 91 | 67 | 4733615 | 6398599 | 3384401 | 114 | 155 | 82 |
| 2018 | both | 7894611 | 8830487 | 6622894 | 100 | 112 | 84 | 8271227 | 11169364 | 5868907 | 106 | 143 | 75 |
|  | male | 4462518 | 5050017 | 3654444 | 121 | 136 | 99 | 3335318 | 4518412 | 2349543 | 92 | 125 | 65 |
|  | female | 3432093 | 3911681 | 2789466 | 81 | 92 | 66 | 4935909 | 6669789 | 3531025 | 116 | 157 | 83 |
| 2019 | both | 8026949 | 9036379 | 6699941 | 100 | 112 | 83 | 8620517 | 11640097 | 6115780 | 107 | 145 | 76 |
|  | male | 4516046 | 5108160 | 3712923 | 119 | 135 | 98 | 3474834 | 4707952 | 2450231 | 93 | 127 | 66 |
|  | female | 3510903 | 4051879 | 2830142 | 80 | 93 | 65 | 5145684 | 6958472 | 3682031 | 118 | 159 | 84 |

Table S2. Numbers and age-standardised rates of YLLs and YLDs of LBMD related fractures(falls) at the global level by sex, 1990-2019.

| year | sex | YLLs number | upper | lower | YLLs rate | upper | lower | YLDs number | upper | lower | YLDs rate | upper | lower |
| --- | --- | --- | --- | --- | --- | --- | --- | --- | --- | --- | --- | --- | --- |
| 1990 | both | 1961468 | 2164011 | 1696516 | 57 | 63 | 49 | 2475321 | 3362221 | 1762409 | 69 | 93 | 49 |
|  | male | 841930 | 939579 | 723552 | 55 | 61 | 48 | 836613 | 1145734 | 596165 | 51 | 69 | 36 |
|  | female | 1119538 | 1280924 | 910835 | 57 | 65 | 46 | 1638707 | 2222133 | 1173433 | 81 | 111 | 58 |
| 1991 | both | 2005982 | 2210620 | 1733248 | 56 | 62 | 48 | 2527688 | 3430952 | 1796613 | 69 | 93 | 49 |
|  | male | 863338 | 971788 | 745772 | 55 | 61 | 48 | 855053 | 1172842 | 609636 | 51 | 69 | 36 |
|  | female | 1142644 | 1308978 | 925283 | 57 | 65 | 46 | 1672635 | 2268099 | 1197697 | 81 | 110 | 58 |
| 1992 | both | 2049734 | 2253238 | 1772314 | 56 | 62 | 48 | 2581842 | 3501952 | 1834123 | 68 | 93 | 49 |
|  | male | 885088 | 997394 | 759231 | 55 | 61 | 48 | 874585 | 1198713 | 622404 | 51 | 69 | 36 |
|  | female | 1164645 | 1325221 | 936136 | 56 | 64 | 45 | 1707257 | 2315253 | 1221637 | 81 | 110 | 58 |
| 1993 | both | 2107890 | 2308097 | 1823158 | 56 | 62 | 49 | 2637491 | 3573452 | 1868338 | 68 | 93 | 48 |
|  | male | 915745 | 1026569 | 788971 | 55 | 61 | 48 | 894662 | 1227535 | 636173 | 51 | 69 | 36 |
|  | female | 1192144 | 1356718 | 973666 | 56 | 64 | 46 | 1742829 | 2365589 | 1242589 | 81 | 110 | 58 |
| 1994 | both | 2145106 | 2348691 | 1858713 | 56 | 61 | 48 | 2696418 | 3650548 | 1909954 | 68 | 92 | 48 |
|  | male | 939685 | 1049510 | 814122 | 55 | 61 | 48 | 915814 | 1256113 | 650932 | 51 | 69 | 36 |
|  | female | 1205421 | 1369587 | 981975 | 55 | 63 | 45 | 1780604 | 2415469 | 1269239 | 81 | 110 | 58 |
| 1995 | both | 2179361 | 2394054 | 1883375 | 55 | 61 | 48 | 2759428 | 3738581 | 1951223 | 68 | 92 | 48 |
|  | male | 958277 | 1072188 | 834195 | 55 | 61 | 48 | 938270 | 1287196 | 666206 | 51 | 69 | 36 |
|  | female | 1221084 | 1380237 | 992029 | 55 | 62 | 45 | 1821158 | 2469811 | 1295346 | 81 | 110 | 57 |
| 1996 | both | 2222170 | 2434350 | 1919109 | 55 | 60 | 47 | 2830144 | 3835316 | 2002025 | 68 | 92 | 48 |
|  | male | 980824 | 1091774 | 846492 | 55 | 61 | 48 | 964034 | 1322380 | 684784 | 51 | 69 | 36 |
|  | female | 1241346 | 1406091 | 1001995 | 54 | 62 | 44 | 1866110 | 2532820 | 1328856 | 81 | 110 | 58 |
| 1997 | both | 2282768 | 2509317 | 1976924 | 55 | 61 | 47 | 2909630 | 3941534 | 2057512 | 69 | 93 | 49 |
|  | male | 1006055 | 1131266 | 872306 | 55 | 61 | 48 | 993522 | 1362984 | 704814 | 51 | 70 | 36 |
|  | female | 1276712 | 1448434 | 1029205 | 55 | 62 | 44 | 1916108 | 2603748 | 1364339 | 81 | 110 | 58 |
| 1998 | both | 2335129 | 2554717 | 2001698 | 55 | 60 | 47 | 2992382 | 4057116 | 2116064 | 69 | 93 | 49 |
|  | male | 1025281 | 1140795 | 884251 | 55 | 60 | 48 | 1024298 | 1405178 | 725650 | 52 | 70 | 37 |
|  | female | 1309848 | 1480779 | 1059010 | 55 | 62 | 44 | 1968085 | 2676388 | 1401224 | 81 | 110 | 58 |
| 1999 | both | 2386881 | 2618190 | 2049894 | 55 | 60 | 47 | 3074603 | 4166992 | 2171110 | 69 | 93 | 49 |
|  | male | 1046248 | 1154538 | 906043 | 55 | 60 | 47 | 1054631 | 1445569 | 746743 | 52 | 71 | 37 |
|  | female | 1340632 | 1515825 | 1081786 | 54 | 62 | 44 | 2019972 | 2744698 | 1436824 | 81 | 110 | 58 |
| 2000 | both | 2453475 | 2685872 | 2103997 | 55 | 60 | 47 | 3152512 | 4275110 | 2227378 | 69 | 93 | 49 |
|  | male | 1081557 | 1192298 | 933717 | 55 | 60 | 48 | 1082522 | 1483891 | 765502 | 52 | 71 | 37 |
|  | female | 1371918 | 1542716 | 1107375 | 54 | 61 | 44 | 2069990 | 2815229 | 1473098 | 81 | 111 | 58 |
| 2001 | both | 2537348 | 2783244 | 2169453 | 55 | 61 | 47 | 3212115 | 4357460 | 2266711 | 68 | 93 | 48 |
|  | male | 1119229 | 1239723 | 967798 | 55 | 61 | 48 | 1102682 | 1509927 | 779417 | 52 | 70 | 37 |
|  | female | 1418120 | 1597779 | 1138793 | 55 | 62 | 44 | 2109433 | 2867427 | 1499395 | 81 | 110 | 57 |
| 2002 | both | 2636276 | 2885445 | 2243253 | 56 | 61 | 47 | 3249773 | 4409732 | 2291465 | 68 | 91 | 48 |
|  | male | 1162785 | 1273810 | 998186 | 56 | 61 | 48 | 1114291 | 1525437 | 786781 | 51 | 69 | 36 |
|  | female | 1473492 | 1666591 | 1168845 | 55 | 63 | 44 | 2135482 | 2902555 | 1515773 | 80 | 108 | 57 |
| 2003 | both | 2742450 | 3010098 | 2323589 | 57 | 63 | 48 | 3278675 | 4450723 | 2312698 | 66 | 90 | 47 |
|  | male | 1212167 | 1324577 | 1035501 | 57 | 62 | 48 | 1121938 | 1537308 | 791919 | 50 | 68 | 35 |
|  | female | 1530282 | 1726729 | 1197538 | 56 | 63 | 44 | 2156737 | 2930094 | 1531367 | 78 | 107 | 56 |
| 2004 | both | 2809422 | 3107493 | 2338431 | 56 | 63 | 47 | 3316261 | 4501704 | 2337980 | 65 | 89 | 46 |
|  | male | 1242679 | 1361534 | 1047490 | 57 | 62 | 48 | 1132191 | 1551282 | 798637 | 49 | 67 | 35 |
|  | female | 1566743 | 1774125 | 1206249 | 56 | 63 | 43 | 2184070 | 2968705 | 1549302 | 77 | 105 | 55 |
| 2005 | both | 2931387 | 3255364 | 2416078 | 57 | 64 | 47 | 3378084 | 4584234 | 2379860 | 65 | 88 | 46 |
|  | male | 1294900 | 1424035 | 1086278 | 57 | 63 | 48 | 1150967 | 1579381 | 811506 | 49 | 67 | 35 |
|  | female | 1636488 | 1847646 | 1255200 | 57 | 64 | 43 | 2227117 | 3025527 | 1577673 | 77 | 104 | 54 |
| 2006 | both | 3017449 | 3347169 | 2463099 | 57 | 64 | 47 | 3463942 | 4703847 | 2442185 | 65 | 88 | 46 |
|  | male | 1327716 | 1457789 | 1106877 | 57 | 63 | 48 | 1178187 | 1616409 | 830241 | 49 | 66 | 34 |
|  | female | 1689733 | 1911340 | 1284048 | 57 | 65 | 43 | 2285755 | 3108205 | 1620954 | 77 | 104 | 54 |
| 2007 | both | 3101922 | 3443482 | 2530784 | 57 | 64 | 47 | 3561817 | 4838548 | 2510458 | 64 | 87 | 45 |
|  | male | 1367002 | 1509807 | 1135289 | 57 | 63 | 48 | 1209164 | 1661015 | 852458 | 49 | 66 | 34 |
|  | female | 1734919 | 1966969 | 1306363 | 57 | 65 | 43 | 2352653 | 3201489 | 1668372 | 77 | 104 | 54 |
| 2008 | both | 3186575 | 3551750 | 2612171 | 57 | 64 | 47 | 3666581 | 4983166 | 2585735 | 64 | 87 | 45 |
|  | male | 1412676 | 1556892 | 1178851 | 57 | 63 | 48 | 1241814 | 1705499 | 876179 | 49 | 66 | 34 |
|  | female | 1773899 | 2016917 | 1348729 | 56 | 64 | 43 | 2424766 | 3302230 | 1721323 | 77 | 104 | 54 |
| 2009 | both | 3199120 | 3552887 | 2625474 | 55 | 62 | 46 | 3777975 | 5133145 | 2666236 | 64 | 87 | 46 |
|  | male | 1430758 | 1574121 | 1189607 | 56 | 62 | 47 | 1276569 | 1752080 | 900431 | 48 | 66 | 34 |
|  | female | 1768362 | 2001365 | 1353242 | 55 | 62 | 42 | 2501405 | 3410001 | 1777125 | 77 | 105 | 54 |
| 2010 | both | 3265756 | 3626289 | 2699828 | 55 | 61 | 45 | 3894536 | 5292176 | 2746728 | 65 | 88 | 46 |
|  | male | 1464611 | 1618880 | 1220317 | 56 | 62 | 47 | 1313136 | 1801954 | 926016 | 48 | 66 | 34 |
|  | female | 1801145 | 2037161 | 1403942 | 54 | 61 | 42 | 2581400 | 3520712 | 1833461 | 77 | 105 | 55 |
| 2011 | both | 3357299 | 3728165 | 2785796 | 55 | 61 | 45 | 4017964 | 5458840 | 2834528 | 65 | 88 | 46 |
|  | male | 1500503 | 1657382 | 1245466 | 55 | 61 | 46 | 1352606 | 1855993 | 955365 | 48 | 66 | 34 |
|  | female | 1856797 | 2107474 | 1453737 | 54 | 61 | 43 | 2665358 | 3634012 | 1892816 | 77 | 105 | 55 |
| 2012 | both | 3425014 | 3792458 | 2835441 | 54 | 60 | 45 | 4152193 | 5646108 | 2931551 | 65 | 88 | 46 |
|  | male | 1536206 | 1693427 | 1276697 | 55 | 61 | 46 | 1396598 | 1915190 | 987010 | 49 | 66 | 34 |
|  | female | 1888808 | 2131251 | 1470937 | 53 | 60 | 42 | 2755595 | 3758006 | 1959247 | 77 | 105 | 55 |
| 2013 | both | 3548682 | 3936209 | 2934383 | 55 | 61 | 45 | 4295420 | 5840250 | 3032928 | 65 | 88 | 46 |
|  | male | 1588301 | 1759813 | 1315896 | 56 | 62 | 46 | 1443296 | 1979251 | 1019677 | 49 | 66 | 34 |
|  | female | 1960381 | 2214164 | 1527970 | 54 | 61 | 42 | 2852124 | 3888529 | 2026985 | 78 | 106 | 55 |
| 2014 | both | 3653769 | 4063977 | 3007149 | 55 | 61 | 45 | 4449382 | 6046697 | 3143119 | 65 | 89 | 46 |
|  | male | 1628491 | 1812461 | 1339136 | 56 | 62 | 46 | 1493230 | 2045157 | 1052859 | 49 | 66 | 34 |
|  | female | 2025279 | 2294596 | 1585567 | 54 | 61 | 42 | 2956152 | 4030714 | 2102986 | 78 | 106 | 56 |
| 2015 | both | 3790860 | 4225813 | 3090370 | 55 | 61 | 45 | 4615048 | 6270693 | 3257199 | 66 | 89 | 46 |
|  | male | 1676177 | 1869083 | 1385597 | 55 | 62 | 46 | 1546284 | 2117124 | 1090300 | 49 | 67 | 35 |
|  | female | 2114683 | 2388360 | 1633949 | 54 | 62 | 42 | 3068764 | 4184504 | 2180307 | 79 | 107 | 56 |
| 2016 | both | 3904281 | 4366092 | 3179244 | 55 | 61 | 45 | 4865390 | 6626830 | 3441603 | 67 | 92 | 48 |
|  | male | 1723380 | 1934124 | 1416117 | 55 | 62 | 45 | 1620345 | 2215705 | 1142204 | 50 | 68 | 35 |
|  | female | 2180901 | 2477410 | 1675075 | 54 | 62 | 42 | 3245045 | 4421288 | 2311469 | 81 | 110 | 58 |
| 2017 | both | 4000170 | 4502628 | 3228764 | 54 | 61 | 44 | 5137962 | 7011212 | 3647787 | 69 | 94 | 49 |
|  | male | 1760790 | 2007445 | 1415062 | 55 | 62 | 44 | 1703389 | 2327692 | 1201096 | 51 | 70 | 36 |
|  | female | 2239379 | 2562404 | 1714336 | 54 | 62 | 41 | 3434573 | 4675686 | 2454291 | 83 | 113 | 59 |
| 2018 | both | 4117503 | 4658705 | 3325152 | 54 | 62 | 44 | 5360819 | 7320495 | 3808100 | 70 | 95 | 50 |
|  | male | 1805658 | 2050625 | 1474458 | 54 | 62 | 45 | 1778626 | 2427530 | 1256392 | 52 | 70 | 36 |
|  | female | 2311845 | 2678283 | 1764798 | 54 | 63 | 41 | 3582193 | 4878270 | 2562534 | 84 | 114 | 60 |
| 2019 | both | 4228800 | 4817179 | 3418417 | 54 | 62 | 44 | 5579664 | 7625209 | 3969171 | 71 | 96 | 50 |
|  | male | 1848491 | 2106739 | 1507531 | 54 | 61 | 44 | 1855954 | 2535199 | 1310899 | 52 | 71 | 37 |
|  | female | 2380309 | 2793715 | 1802935 | 54 | 64 | 41 | 3723711 | 5079729 | 2667144 | 85 | 116 | 61 |

Table S3. DALYs and deaths number and age-standardized rate of fractures due to LBMD by GBD region, 2019.

| region | DALYs number | DALYs rate | death number | death rate | SDI classification |
| --- | --- | --- | --- | --- | --- |
| Afghanistan | 5791.51 | 52.97 | 154.16 | 2.29 | low SDI |
| Albania | 3496.97 | 83.59 | 33.27 | 0.82 | middle SDI |
| Algeria | 17936.72 | 58.67 | 382.73 | 1.77 | middle SDI |
| American Samoa | 32.86 | 78.06 | 1.03 | 3.02 | high-middle SDI |
| Andorra | 308.95 | 202.14 | 10.89 | 6.32 | high SDI |
| Angola | 6455.20 | 80.00 | 228.88 | 4.07 | low-middle SDI |
| Antigua and Barbuda | 41.41 | 45.25 | 1.42 | 1.81 | high-middle SDI |
| Argentina | 37750.43 | 69.61 | 855.13 | 1.52 | high-middle SDI |
| Armenia | 2280.71 | 56.25 | 32.57 | 0.84 | middle SDI |
| Australia | 71878.28 | 160.51 | 1949.70 | 3.83 | high SDI |
| Austria | 30433.32 | 158.05 | 832.13 | 3.68 | high SDI |
| Azerbaijan | 4738.19 | 50.96 | 42.17 | 0.66 | middle SDI |
| Bahamas | 192.34 | 55.23 | 6.75 | 2.22 | high-middle SDI |
| Bahrain | 444.40 | 45.73 | 4.56 | 1.15 | high-middle SDI |
| Bangladesh | 51347.13 | 41.76 | 1167.47 | 1.19 | low-middle SDI |
| Barbados | 224.43 | 45.90 | 8.47 | 1.76 | high-middle SDI |
| Belarus | 21390.35 | 137.25 | 245.81 | 1.54 | high-middle SDI |
| Belgium | 52300.24 | 209.19 | 1479.63 | 4.89 | high SDI |
| Belize | 130.35 | 51.96 | 4.57 | 2.05 | low-middle SDI |
| Benin | 3053.71 | 78.60 | 128.86 | 4.06 | low SDI |
| Bermuda | 67.26 | 49.64 | 2.26 | 1.54 | high SDI |
| Bhutan | 914.99 | 188.88 | 41.20 | 9.70 | high SDI |
| Bolivia (Plurinational State of) | 7182.28 | 87.61 | 250.91 | 3.61 | low-middle SDI |
| Bosnia and Herzegovina | 6327.09 | 109.53 | 95.13 | 1.75 | high-middle SDI |
| Botswana | 543.62 | 46.06 | 13.91 | 1.63 | middle SDI |
| Brazil | 258807.53 | 112.89 | 7836.56 | 3.62 | middle SDI |
| Brunei Darussalam | 278.00 | 109.16 | 4.41 | 2.97 | high SDI |
| Bulgaria | 14398.45 | 103.76 | 170.19 | 1.17 | high-middle SDI |
| Burkina Faso | 6413.22 | 90.73 | 270.98 | 4.79 | low SDI |
| Burundi | 2796.53 | 80.16 | 97.75 | 3.88 | low SDI |
| Cabo Verde | 296.44 | 71.46 | 14.65 | 3.50 | low-middle SDI |
| Cambodia | 18049.66 | 181.69 | 684.90 | 8.55 | low-middle SDI |
| Cameroon | 7878.24 | 87.12 | 321.51 | 4.53 | low-middle SDI |
| Canada | 108253.33 | 144.89 | 3576.62 | 4.34 | high SDI |
| Central African Republic | 1166.87 | 73.09 | 40.64 | 3.71 | low SDI |
| Chad | 3466.10 | 77.55 | 147.03 | 4.08 | low SDI |
| Chile | 23900.65 | 100.10 | 679.60 | 2.89 | high-middle SDI |
| China | 1839375.07 | 104.83 | 56639.13 | 4.07 | middle SDI |
| Colombia | 27202.77 | 50.81 | 645.55 | 1.15 | middle SDI |
| Comoros | 311.17 | 72.34 | 11.72 | 3.24 | high SDI |
| Congo | 1423.48 | 72.85 | 53.56 | 3.84 | low-middle SDI |
| Cook Islands | 14.81 | 61.56 | 0.34 | 1.54 | high-middle SDI |
| Costa Rica | 4597.77 | 89.23 | 189.43 | 3.52 | middle SDI |
| Croatia | 20937.64 | 226.51 | 648.38 | 6.85 | high-middle SDI |
| Cuba | 34919.00 | 170.48 | 1843.41 | 8.47 | middle SDI |
| Cyprus | 2732.37 | 153.19 | 70.08 | 4.59 | high SDI |
| Czechia | 35828.88 | 168.26 | 928.07 | 4.15 | high SDI |
| Côte d'Ivoire | 6433.66 | 82.65 | 251.10 | 4.27 | low SDI |
| Democratic People's Republic of Korea | 16762.03 | 56.17 | 708.94 | 2.80 | low-middle SDI |
| Democratic Republic of the Congo | 21509.33 | 79.19 | 820.35 | 3.97 | low SDI |
| Denmark | 16085.80 | 133.35 | 517.53 | 3.79 | high SDI |
| Djibouti | 333.56 | 75.22 | 9.93 | 3.45 | high SDI |
| Dominica | 40.14 | 44.17 | 1.60 | 1.76 | high-middle SDI |
| Dominican Republic | 4375.35 | 50.12 | 163.17 | 2.05 | low-middle SDI |
| Ecuador | 11723.36 | 81.70 | 365.71 | 2.93 | middle SDI |
| Egypt | 24235.50 | 41.66 | 430.69 | 1.06 | middle SDI |
| El Salvador | 5177.65 | 83.76 | 188.18 | 2.80 | low-middle SDI |
| Equatorial Guinea | 252.97 | 69.38 | 10.05 | 3.60 | middle SDI |
| Eritrea | 1662.43 | 85.28 | 51.81 | 4.06 | low SDI |
| Estonia | 2831.55 | 112.01 | 39.13 | 1.37 | high SDI |
| Eswatini | 215.19 | 43.63 | 5.81 | 1.67 | low-middle SDI |
| Ethiopia | 29266.68 | 87.37 | 1204.85 | 4.48 | low SDI |
| Fiji | 341.20 | 55.91 | 7.80 | 2.02 | middle SDI |
| Finland | 26729.17 | 208.63 | 684.89 | 4.49 | high SDI |
| France | 279998.86 | 178.20 | 9320.90 | 4.60 | high SDI |
| Gabon | 665.08 | 79.10 | 26.80 | 3.96 | middle SDI |
| Gambia | 741.78 | 93.73 | 34.22 | 5.10 | low SDI |
| Georgia | 6241.04 | 105.27 | 100.91 | 1.57 | high SDI |
| Germany | 297944.30 | 143.04 | 8541.97 | 3.53 | high SDI |
| Ghana | 10786.30 | 86.80 | 430.07 | 4.35 | low-middle SDI |
| Greece | 21275.13 | 89.18 | 374.06 | 1.19 | high-middle SDI |
| Greenland | 155.06 | 268.97 | 4.29 | 9.15 | high-middle SDI |
| Grenada | 65.30 | 65.35 | 2.24 | 2.64 | middle SDI |
| Guam | 104.72 | 55.84 | 2.72 | 1.46 | high SDI |
| Guatemala | 11031.27 | 106.47 | 347.61 | 3.92 | low-middle SDI |
| Guinea | 3671.90 | 78.85 | 165.73 | 4.07 | low SDI |
| Guinea-Bissau | 492.93 | 91.18 | 19.65 | 4.80 | low SDI |
| Guyana | 554.27 | 105.46 | 20.54 | 4.90 | middle SDI |
| Haiti | 3565.30 | 64.74 | 138.32 | 3.35 | low SDI |
| Honduras | 3345.85 | 58.24 | 84.75 | 1.74 | low-middle SDI |
| Hungary | 35354.51 | 177.16 | 928.32 | 4.37 | high-middle SDI |
| Iceland | 731.97 | 123.77 | 18.81 | 2.63 | high SDI |
| India | 2510287.65 | 258.50 | 93674.94 | 11.47 | low-middle SDI |
| Indonesia | 177800.34 | 103.71 | 6295.12 | 4.88 | middle SDI |
| Iran (Islamic Republic of) | 36925.65 | 53.89 | 851.10 | 1.46 | middle SDI |
| Iraq | 10153.26 | 45.46 | 150.15 | 0.91 | middle SDI |
| Ireland | 8812.48 | 117.16 | 176.59 | 2.24 | high SDI |
| Israel | 10820.30 | 91.18 | 252.40 | 1.87 | high-middle SDI |
| Italy | 194283.28 | 118.31 | 6488.00 | 3.18 | high-middle SDI |
| Jamaica | 1232.73 | 39.39 | 43.62 | 1.20 | middle SDI |
| Japan | 323094.28 | 84.63 | 7258.14 | 1.33 | high SDI |
| Jordan | 2846.82 | 47.50 | 47.77 | 1.29 | high-middle SDI |
| Kazakhstan | 13166.21 | 76.56 | 145.96 | 1.00 | high-middle SDI |
| Kenya | 14174.61 | 85.41 | 541.47 | 4.35 | low-middle SDI |
| Kiribati | 24.37 | 46.53 | 0.54 | 1.87 | low-middle SDI |
| Kuwait | 1458.71 | 49.82 | 18.38 | 0.91 | high SDI |
| Kyrgyzstan | 2432.19 | 52.82 | 25.31 | 0.65 | low-middle SDI |
| Lao People's Democratic Republic | 3087.90 | 83.16 | 107.17 | 3.67 | low-middle SDI |
| Latvia | 4822.63 | 125.05 | 82.89 | 1.92 | high SDI |
| Lebanon | 2493.27 | 49.22 | 61.33 | 1.31 | high-middle SDI |
| Lesotho | 570.09 | 52.56 | 17.36 | 2.16 | low-middle SDI |
| Liberia | 1217.85 | 75.99 | 53.01 | 4.09 | low SDI |
| Libya | 2860.77 | 57.17 | 63.04 | 1.52 | high-middle SDI |
| Lithuania | 8804.92 | 155.49 | 153.15 | 2.43 | high SDI |
| Luxembourg | 1708.71 | 158.86 | 47.27 | 3.89 | high SDI |
| Madagascar | 5375.08 | 63.37 | 150.17 | 2.72 | low SDI |
| Malawi | 5239.94 | 90.28 | 198.14 | 4.32 | low SDI |
| Malaysia | 17641.07 | 75.45 | 581.53 | 2.97 | high-middle SDI |
| Maldives | 194.12 | 72.16 | 6.25 | 2.74 | low-middle SDI |
| Mali | 6771.34 | 99.51 | 295.76 | 5.30 | low SDI |
| Malta | 1258.74 | 134.34 | 27.67 | 2.62 | high-middle SDI |
| Marshall Islands | 19.22 | 75.38 | 0.51 | 3.43 | low-middle SDI |
| Mauritania | 1228.50 | 69.96 | 53.34 | 3.53 | low-middle SDI |
| Mauritius | 829.78 | 48.60 | 18.56 | 1.18 | high-middle SDI |
| Mexico | 103964.71 | 92.86 | 2853.35 | 2.79 | middle SDI |
| Micronesia (Federated States of) | 43.69 | 82.85 | 1.33 | 3.84 | low-middle SDI |
| Monaco | 112.06 | 111.31 | 2.45 | 1.87 | high SDI |
| Mongolia | 2293.05 | 92.07 | 27.93 | 1.38 | low-middle SDI |
| Montenegro | 1213.66 | 127.89 | 20.35 | 2.35 | high-middle SDI |
| Morocco | 24467.70 | 88.57 | 710.97 | 3.31 | low-middle SDI |
| Mozambique | 8757.90 | 102.02 | 308.03 | 4.82 | low SDI |
| Myanmar | 55156.25 | 138.15 | 2056.46 | 6.18 | low-middle SDI |
| Namibia | 593.16 | 46.70 | 19.20 | 1.81 | middle SDI |
| Nauru | 2.47 | 80.38 | 0.05 | 3.32 | middle SDI |
| Nepal | 37633.76 | 186.52 | 1179.35 | 7.02 | low SDI |
| Netherlands | 61700.81 | 168.02 | 2216.35 | 5.62 | high SDI |
| New Zealand | 13778.53 | 168.02 | 331.28 | 3.55 | high SDI |
| Nicaragua | 3231.47 | 85.80 | 119.60 | 3.94 | low-middle SDI |
| Niger | 5921.17 | 103.47 | 238.01 | 5.51 | low SDI |
| Nigeria | 52799.86 | 78.59 | 2266.79 | 4.18 | low-middle SDI |
| Niue | 1.54 | 72.40 | 0.05 | 2.59 | high-middle SDI |
| North Macedonia | 3261.27 | 112.18 | 58.16 | 2.62 | high-middle SDI |
| Northern Mariana Islands | 39.46 | 98.29 | 0.95 | 3.64 | high-middle SDI |
| Norway | 20159.64 | 187.93 | 694.97 | 5.59 | high SDI |
| Oman | 1252.73 | 93.67 | 25.75 | 3.83 | high-middle SDI |
| Pakistan | 52254.89 | 57.24 | 1667.78 | 2.48 | low SDI |
| Palau | 22.44 | 135.24 | 0.61 | 5.45 | high-middle SDI |
| Palestine | 1428.83 | 68.28 | 31.83 | 2.12 | low-middle SDI |
| Panama | 2040.67 | 48.60 | 51.90 | 1.18 | middle SDI |
| Papua New Guinea | 4996.62 | 161.08 | 186.27 | 9.07 | low SDI |
| Paraguay | 4000.40 | 74.94 | 129.95 | 2.53 | middle SDI |
| Peru | 16772.04 | 52.16 | 438.62 | 1.34 | middle SDI |
| Philippines | 45051.11 | 66.71 | 1317.54 | 2.46 | middle SDI |
| Poland | 105513.42 | 150.35 | 2372.67 | 3.14 | high-middle SDI |
| Portugal | 21677.44 | 84.75 | 559.58 | 1.85 | high-middle SDI |
| Puerto Rico | 5356.69 | 71.24 | 164.69 | 1.85 | high SDI |
| Qatar | 761.85 | 78.09 | 6.43 | 2.44 | high SDI |
| Republic of Korea | 105639.37 | 121.39 | 2195.51 | 2.77 | high SDI |
| Republic of Moldova | 5411.19 | 94.47 | 61.34 | 1.06 | middle SDI |
| Romania | 41935.96 | 116.49 | 465.74 | 1.23 | high-middle SDI |
| Russian Federation | 258036.65 | 111.97 | 3053.94 | 1.32 | high-middle SDI |
| Rwanda | 4022.74 | 86.82 | 140.01 | 4.09 | low SDI |
| Saint Kitts and Nevis | 38.51 | 70.75 | 1.26 | 2.90 | high-middle SDI |
| Saint Lucia | 94.33 | 45.62 | 3.48 | 1.83 | middle SDI |
| Saint Vincent and the Grenadines | 85.59 | 67.77 | 3.40 | 3.05 | middle SDI |
| Samoa | 95.83 | 73.60 | 3.36 | 3.01 | middle SDI |
| San Marino | 79.52 | 114.48 | 1.96 | 2.19 | high SDI |
| Sao Tome and Principe | 117.36 | 134.60 | 5.06 | 7.05 | low-middle SDI |
| Saudi Arabia | 36012.77 | 196.97 | 566.21 | 4.97 | high-middle SDI |
| Senegal | 5213.36 | 85.19 | 232.58 | 4.51 | low SDI |
| Serbia | 17069.36 | 111.18 | 283.36 | 1.96 | high-middle SDI |
| Seychelles | 58.36 | 55.29 | 1.53 | 1.65 | high-middle SDI |
| Sierra Leone | 2242.16 | 77.31 | 96.26 | 4.04 | low SDI |
| Singapore | 5640.59 | 73.50 | 67.11 | 0.96 | high SDI |
| Slovakia | 15871.59 | 175.20 | 305.91 | 3.44 | high SDI |
| Slovenia | 10176.13 | 215.28 | 296.95 | 5.46 | high SDI |
| Solomon Islands | 510.17 | 213.43 | 15.50 | 9.68 | low SDI |
| Somalia | 3640.28 | 73.56 | 110.10 | 3.43 | low SDI |
| South Africa | 12750.30 | 30.93 | 300.15 | 0.90 | middle SDI |
| South Sudan | 2008.25 | 63.59 | 67.93 | 2.90 | low SDI |
| Spain | 112078.33 | 110.30 | 2262.39 | 1.74 | high-middle SDI |
| Sri Lanka | 25043.64 | 110.58 | 837.91 | 4.40 | middle SDI |
| Sudan | 9971.33 | 56.61 | 225.48 | 1.64 | low-middle SDI |
| Suriname | 323.20 | 57.92 | 12.57 | 2.43 | middle SDI |
| Sweden | 37902.62 | 161.85 | 1155.30 | 4.11 | high SDI |
| Switzerland | 37808.34 | 193.31 | 1201.33 | 5.05 | high SDI |
| Syrian Arab Republic | 6467.51 | 55.93 | 95.14 | 1.33 | middle SDI |
| Taiwan (Province of China) | 23735.51 | 59.64 | 806.44 | 1.98 | high SDI |
| Tajikistan | 2957.51 | 58.43 | 32.38 | 0.92 | low-middle SDI |
| Thailand | 67060.44 | 66.17 | 1809.74 | 1.84 | middle SDI |
| Timor-Leste | 568.32 | 80.53 | 19.66 | 3.48 | low-middle SDI |
| Togo | 2172.21 | 80.38 | 81.94 | 4.06 | low SDI |
| Tokelau | 0.83 | 67.71 | 0.03 | 2.66 | middle SDI |
| Tonga | 45.31 | 58.71 | 1.63 | 2.19 | middle SDI |
| Trinidad and Tobago | 693.33 | 38.68 | 20.14 | 1.18 | high-middle SDI |
| Tunisia | 6400.66 | 53.52 | 146.09 | 1.41 | middle SDI |
| Turkey | 67191.26 | 80.78 | 2251.72 | 2.91 | high-middle SDI |
| Turkmenistan | 1817.29 | 46.46 | 15.38 | 0.50 | middle SDI |
| Tuvalu | 6.46 | 74.18 | 0.22 | 3.17 | low-middle SDI |
| Uganda | 9197.42 | 82.49 | 339.25 | 3.98 | low SDI |
| Ukraine | 80578.89 | 109.61 | 777.74 | 1.05 | high-middle SDI |
| United Arab Emirates | 3307.91 | 55.98 | 36.10 | 1.16 | high SDI |
| United Kingdom | 169926.06 | 128.38 | 4549.05 | 2.98 | high SDI |
| United Republic of Tanzania | 17318.56 | 85.37 | 645.46 | 3.97 | low SDI |
| United States of America | 819444.96 | 139.09 | 22173.99 | 3.39 | high SDI |
| United States Virgin Islands | 101.62 | 59.67 | 3.70 | 2.49 | high-middle SDI |
| Uruguay | 5058.89 | 86.94 | 162.67 | 2.35 | middle SDI |
| Uzbekistan | 11333.66 | 55.76 | 106.22 | 0.89 | middle SDI |
| Vanuatu | 99.56 | 70.19 | 3.17 | 3.18 | low-middle SDI |
| Venezuela (Bolivarian Republic of) | 17602.80 | 62.80 | 537.63 | 2.01 | low-middle SDI |
| Viet Nam | 114018.90 | 146.01 | 5413.33 | 7.83 | middle SDI |
| Yemen | 7342.48 | 60.30 | 170.48 | 1.92 | low SDI |
| Zambia | 4909.30 | 95.59 | 171.73 | 4.55 | low-middle SDI |
| Zimbabwe | 3877.41 | 73.06 | 151.23 | 3.86 | low-middle SDI |
